# Developing an ‘Insider Language Index’ as a composite measure to detect insider threat

**DOI:** 10.1080/13218719.2025.2486081

**Published:** 2025-05-29

**Authors:** Natasha G. Martlew, Linden J. Ball, Coral J. Dando, Thomas C. Ormerod, Paul J. Taylor, Tarek Menacere, Alexandra L. Sandham, Beth H. Richardson

**Affiliations:** a School of Psychology & Humanities, University of Central Lancashire, Preston, UK; b Department of Psychology, University of Westminster, London, UK; c Thomas C. Ormerod, School of Psychology, University of Sussex, Falmer, UK; d Department of Psychology, Lancaster University, Lancaster, UK; e Department of Psychology, University of Gloucestershire, Cheltenham, UK

**Keywords:** insider threat, language use, threat detection, mitigation, deception, cognitive processing, negative emotion, pronoun use

## Abstract

Of all the issues that confront modern organisations, insider threat is one of the most challenging in terms of impact and mitigation. Typically, research has focused on defining what insider threat is and determining how such threats can be detected through technology. We suggest that technological approaches to insider-threat detection can be complemented through a greater focus on investigating the linguistic behaviours associated with insider activity. Research has highlighted that an individual’s use of language offers a potential means of identifying insiders. Using Linguistic Inquiry and Word Count, this study analysed the language used by insiders and non-insiders during workplace interviews. Results revealed that, compared to non-insiders, insiders used significantly more words relating to cognitive processing, significantly more self-referential terms, and significantly more negative emotion words. Based on these findings, a generalisable Insider Language Index (ILI) was developed that has the potential to support insider detection in organisational contexts.

## Introduction

Insiders are often current or former employees, business partners, or service providers who have legitimate access to a company’s information and steal or compromise such information for malicious reasons, including for revenge and personal gain (Duncan et al., 2015). The threat that insiders pose to organisations continues to be a significant concern (Schultz, 2002). For example, in 2016, a former employee at Google who became unhappy with his engineering role on Google’s self-driving car project (Waymo), stole 14,000 Google files relating to a $1.1 billion technology project, and provided these trade secrets to Uber, his new employer. This data breach demonstrates the extent to which undetected insider attacks can result in significant and detrimental losses to organisations, underscoring the need to develop effective detection and mitigation approaches.

To address the issue of insider threat detection, the present research sought to investigate the linguistic factors associated with an attack. Such knowledge can be used to inform the development of methods to assist employers to mitigate the potential damage that could be caused by insiders. One of the principal obstacles in researching the detection and prevention of insider attacks is that relatively few psychological studies in this field have been conducted. Of the studies that have been undertaken, most have been designed to solve specific problems within specific organisations, and thus there are very few studies to guide generic approaches to the wider problem of insider threat detection (Legg et al., 2015; Maloof & Stephens, 2007). As such, in the present research we aimed to develop a generalisable Insider Language Index (ILI) that could be deployed across many real-world contexts to assist in detecting insiders, reducing the potential damage that could occur as a result of an attack.

### Technological approaches to detecting and mitigating insider threat

Typically, research has focused on defining what insider threat is, as well as determining how these types of threats can be predicted, detected, and prevented through technology, such as by tracking system-access and implementing countermeasures (Nurse et al., 2014). For example, Salem and Stolfo (2009) used decoy documents to identify malicious intent during masquerade attacks (e.g. involving use of a stolen identity). The method for monitoring and detecting insiders carrying out such attacks used baited decoy documents to deceive, confuse, and confound attackers, forcing them to expend much more effort to discern real from fabricated information. This approach made it difficult for insiders to avoid detection as the decoys were difficult to distinguish from authentic documents. Any alert generated by the decoy was an indication of insider activity. Although these system-based approaches are useful to aid our understanding of insider attacks, such technological approaches are usually only useful post-attack, once a system has been accessed (Brdiczka et al., 2012).

There are a handful of studies that have considered the human element of insider attacks. For example, Tugular and Spafford (1997) proposed a model of insider attacks, which assumed that insider activity is a function of personal characteristics, knowledge, motivation, abilities, rights, and responsibilities within an organisation. The authors noted that insider attacks are more likely to occur in the presence of a breakdown of authority within an organisation (Tugular & Spafford, 1997). Although this model offers a useful starting point, it has little value in terms of helping organisations reduce the frequency or damage caused by insider attacks. Understanding the behavioural characteristics of insiders is arguably far more useful for detecting an attack, including at an early stage so as to curtail further malicious activity. However, because of a current lack of research in this area, countless insider attacks fail to be detected (Legg et al., 2015). Critically, technological approaches typically overlook the central role of the human in insider activity, and more specifically, the linguistic, behavioural, and psychological factors associated with an attack (Taylor et al., 2013). Such factors need to be explored to broaden our understanding of how insiders think, act, and behave, which can in turn aid in the early detection of attacks.

### Language and deception

Although there is a lack of research investigating the importance of language use in insider attacks, a broader literature suggests that language cues may be useful indicators of truth and lies in deception detection (e.g. Dando et al., 2024; Taylor et al., 2013). For example, McCornack (1992) proposed Information Manipulation Theory (IMT), which posits that in ordinary conversations, individuals monitor the information that they disclose across four dimensions: amount (the quantity of information provided), veracity (the quality of information presented), relevance (the relevance of information within conversational contributions), and clarity (the clarity of the information provided within messages). When engaging in deception, individuals covertly alter the information that is disclosed with regard to these four dimensions. In turn, listeners are misled by their belief that speakers are behaving in a cooperative manner (McCornack, 1992). IMT suggests that the information within deceptive messages varies in systematic and identifiable ways and thus can be detected through a focus on objective language cues. The claims of IMT were empirically tested by McCornack (1992). Results revealed that messages containing unclear language, short responses, and irrelevant information significantly influenced their perceived veracity. This theory allows us to understand how an individual can manipulate the language they disclose during a conversation in order to accomplish deceit. In turn, such a theory begins to demonstrate the importance of focusing on the use of language when attempting to detect deception.

Further evidence to demonstrate the potential benefits that a focus on language can have when attempting to detect deceivers can be found in research on Statement Validity Analysis (e.g. Amado et al., 2016), a tool that is used to evaluate the veracity of a testimony. Two of the most common approaches are Criteria Based Content Analysis (CBCA: Berliner & Conte, 1995; Vrij, 2005) and Reality Monitoring (RM; Johnson et al., 1993; Masip et al., 2005). A CBCA evaluation requires an analyst to determine the extent to which a statement shows evidence of 19 linguistic criteria. These include linguistic aspects such as logistical structure, use of emotive language, and descriptions of perceptual details such as sights, sounds, and smells. Like CBCA, RM focuses on linguistic criteria that are more common in truthful than deceptive accounts, which also include perceptual details (e.g. tastes and smells), contextual information (e.g. information about objects or people), and reference to cognitive operations (e.g. thoughts and reasoning; Vrij, 2014).

Research conducted by Porter and Yuille (1996) examined the hypothesis that reliable verbal indicators of deception exist during interrogations. Within this study, participants were informed that they would be engaging in an investigation to address security effectiveness and they were instructed to commit an act such as a theft. They were then asked to provide either a truthful alibi, a partially deceptive account, a false alibi, or a truthful confession regarding the theft, with a monetary incentive for convincing the interrogator of their veracity. Results revealed that three verbal cues (all CBCA criteria) were able to distinguish truthful from deceptive statements: amount of detail reported, information coherence, and admissions of lack of memory. Deceptive suspects were found to provide less detailed accounts, to give less cohesive descriptions, and to be less likely to admit an inability to remember aspects of the target event during interview.

Earlier research conducted by Landry and Brigham (1992) examined the usefulness of CBCA to differentiate truthful from deceptive testimonies in interviews with adults. The study involved 114 students who estimated the veracity of 12 statements, six of which were truthful accounts and six of which involved adults who described a fabricated, traumatic personal experience. Participants viewed the statements via a video recording or a written transcript. Half of the participants were trained in CBCA and half were untrained. Results revealed that trained participants were able to differentiate between truthful and deceptive statements, with accuracy significantly greater than chance level. Trained participants were also significantly more accurate than untrained participants. Such findings from the CBCA literature again underscore that a focus on language may be beneficial when attempting to detect deceivers.

Researchers have additionally sought to detect deception through a focused analysis of the *content* of spoken or written language – for example, by looking for specific word cues or sentence structures that might indicate deception (e.g. Hauch et al., 2015). Some research of this type has shown that, when analysing truthful and deceitful written messages, accuracy rates are at chance level, which suggests that individuals are no better at detecting truth than deceit (Masip et al., 2012). However, when truthful and deceptive messages are analysed using Linguistic Inquiry and Word Count (LIWC; Pennebaker et al., 2007), which examines a text file on a word-by-word basis to calculate the total percentage of words that match a number of linguistic categories, evidence has emerged that certain linguistic characteristics, such as first-person singular pronouns and first-person plural pronouns, are present to a greater extent in deceitful messages. Furthermore, when participants are made aware of the importance of such language categories prior to reading messages, deception-detection accuracy rates have been found to improve from chance level to 68% (Masip et al., 2012). In another study, Burns and Moffitt (2014) used the LIWC automated text analysis software to classify 50 transcripts of truthful and fabricated emergency 911 calls. Results indicated that for deceivers, their attempt to evidence genuine affect resulted in an increased use of negations to signal contradiction or denial (e.g. no, not, never) and more expressions of assent (e.g. agree, approve). In contrast to deceivers, truthful callers actively displayed their felt emotion, as shown in their use of words signalling negative emotion (e.g. hate, worthless, enemy) as well as in their use of anxiety-related words (Burns & Moffitt, 2014). The overall performance of the analysis technique was as high as 84%, which illustrates the potential to use automated linguistic analysis in high-stake situations such as crime investigations.

Although several studies have demonstrated the existence of linguistic differences between truthful and deceptive statements, such differences are not consistent across people and contexts (Levitan et al., 2018; Thompson & Hartwig, 2023; Williams et al., 2014). To help determine the usefulness of linguistic cues for deception detection at the level of the individual, Van der Zee et al. (2022) developed a model tailored to US president Donald Trump, during his first term. When analysing tweets that were checked by an independent third party for factuality, results revealed significant linguistic differences between factually correct and incorrect tweets. As a result of such differences, a quantitative model was developed. Using this model, the authors attempted to predict whether other tweets, not included in the original sample, were factually correct or incorrect. The findings revealed an accuracy rate of 73%. Such research demonstrates the benefit of linguistic analysis when detecting deception at the level of the *individual*, rather than at the group level.

A focus on other language characteristics, such as an increased use of personal pronouns, has also been observed to benefit deception detection (Cohn et al., 2004). Evidence to support this proposal was found in research conducted by Hancock et al. (2007), which computed the percentage of different pronouns used in honest and fabricated conversational transcripts and revealed that fabricated transcripts contained a greater proportion of personal pronouns as compared to honest transcripts. However, not all findings support this trend. Research has suggested that deceptive individuals who are attempting to avoid detection may use fewer personal pronouns to distance themselves from their story (Shapiro, 1996). This ‘distancing strategy’ minimises personal involvement with the content of the message (Newman et al., 2003).

### Language and insider threat

In the context of insider threat detection, there is limited yet growing research to suggest that linguistic characteristics could be used to identify insiders. Research has indicated that the use of first-person plural pronouns (e.g. ‘we’) are less indicative of self-focus and more indicative of a strong sense of community (Bond & Pennebaker, 2012). For example, Cohn et al. (2004) found that, after the 11 September 2001 attack on the Twin Towers, people used a greater proportion of plural pronouns as communities dealt with the incident together. Such findings suggest that a focus on pronoun use may be beneficial when detecting insider activity, as it has been shown to be associated with the self-focus often experienced by an insider.

Negative affect is another language characteristic that has been associated with insider activity. The presence of this characteristic stems from the idea that insiders are often current or former employees who have felt frustrated with their organisation after it has failed to acknowledge an employee’s accomplishments (Giumetti et al., 2013). As a result of such frustration, employees may seek to conduct an insider attack for the purpose of revenge or personal gain. This has been supported by research conducted by Workman and Gathegi (2007), who found that negative affect in the workplace was associated with negative work behaviours such as theft, which has similarities to that of an insider attack as this can involve the individual stealing company information for personal gain.

A third linguistic difference between insiders and non-insiders relates to the cognitive load that insiders may experience when conducting an attack (Vrij et al., 2008). For example, insiders will likely have to exert additional mental effort to be able to maintain both their insider activity and their ordinary work activity. With the increased mental effort required to complete additional tasks, insiders may experience greater cognitive demand and, as such, may use a greater proportion of words relating to cognitive processing (Walczyk et al., 2005). Taken together, such evidence indicates that linguistic characteristics, such as pronoun use, negative affect, and words relating to cognitive processing may be useful cues when attempting to discern insider activity.

Although computational processing systems, such as LIWC (Tausczik & Pennebaker, 2010), have been used to explore linguistic cues to deception detection, such systems are yet to be extensively applied to insider threat detection. One of the few studies that has applied LIWC in insider threat detection was conducted by Taylor et al. (2013). They tested the hypothesis that conducting an insider attack can lead to cognitive and social challenges that may impact an insider’s daily behaviour at work. In light of previous research that has considered individual language use and insider threat, the authors predicted that insiders would use a greater number of first-person singular and second-person pronouns, and fewer first-person plural pronouns, compared to non-insider co-workers. They further predicted that insiders would show increased negative affect and use a greater number of words relating to cognitive processes when compared to non-insider co-workers. Taylor et al.’s (2013) research therefore explored the potential to detect insiders through their language use. Participants took part in a six-hour workplace simulation that required them to examine databases and exchange information as part of an investigation into organised crime. A quarter of the participants were later incentivised to behave as an ‘insider’. During the simulation, participants communicated only via email and these emails were subsequently analysed using LIWC to derive measures of language use. These measures included words that related to current motivations such as the use of personal pronouns (‘I’), cognitive processes, and affect.

The results from Taylor et al.’s (2013) study revealed that insiders, compared to their non-insider counterparts, were more self-focused (signified by an increase in personal pronouns, such as ‘I’ and ‘me’), showed greater negative affect (signified by an increase in words related to negations, such as ‘no’, ‘not’, and ‘can’t’) and showed increased cognitive processing (signified by an increase in words related to cognitive mechanisms; in particular, an increase in the use of discrepancy words such as ‘ought’ and ‘should’). These language changes were not matched by a significant change in the use of first-person plural pronouns. Increases in the use of cognitive processing words can be explained in terms of the cognitive load that is likely to be associated with an insider attack (Spence et al., 2001). This finding has been supported in the deception literature, which has suggested that lying may be more cognitively demanding than telling the truth (Vrij et al., 2008). Taylor et al.’s (2013) findings therefore support the idea that language variables can provide a mechanism for detecting potential insiders. For example, individuals who are looking to detect insider activity within a company could focus on the use of certain language features in employees’ communication.

### Aims of the current study

Here, we aimed to test the generalisability of Taylor et al.’s (2013) findings that insiders might be identified through changes in their language use. Taylor et al. (2013) were concerned only with the language of insiders and non-insiders during *email* communication. Accordingly, several questions arise as to whether differences in language use extend to other communication contexts, such as face-to-face, in-person interactions. It is possible, for example, that the unique features and constraints of email-based communication accentuate the prevalence of certain language features associated with insiders that would not be displayed in other communication contexts.

As a case in point, consider Taylor et al.’s (2013) observation of the increased use of discrepancy words (e.g. ‘ought’ and ‘should’) by insiders relative to non-insiders in their email communication, which is assumed to be indicative of increased cognitive processing. Reid et al. (1997) have shown how such discrepancy words can dominate email-based communication when people are trying to make complex decisions but are only in possession of partial information. Given that insiders might well be preoccupied with engaging in their insider activity (e.g. inappropriately accessing restricted, security-sensitive files) and therefore only be partly focused on the collaborative task at hand, it is possible that their increased use of words such as ‘ought’ and ‘should’ is solely linked to the email-driven nature of the ongoing communication and might not be observed when they are communicating face-to-face.

Indeed, we suggest that there are numerous contexts beside team-based email communication (see Taylor et al., 2013) and computer-mediated communication (see Dando et al., 2024) where it is important to understand whether insiders show the changes in language features identified by Taylor et al. (2013). An example of one such context is an in-person information-gathering investigative interview, which might arise within an organisation or externally following an insider event. The workplace simulation that informed the research reported by Dando et al. (2024) of computer-mediated communication as well as that reported by Taylor et al. (2013) of email-based communication, also produced face-to-face interview data with both insiders and non-insiders, which have not previously been analysed or published. These tactical interviews were conducted directly following the workplace simulation. Nine of the 54 interviews were with insiders who had been tasked to carry out various malicious activities during the simulation, whilst the remainder were with non-insiders. It is this set of interviews that forms the focus of the current research. More specifically, we consider whether language differences reported between insiders and non-insiders in email communication also arise in verbalisations during tactical information-gathering interviews.

Here, we report an analysis of the aforementioned verbal interview data using LIWC (Pennebaker et al., 2007) in order to inform the development of an ‘Insider Language Index’ (ILI) to support differentiating insiders from non-insiders based on patterns of language use. The development of an ILI has the potential to provide an objective and diagnostic method for the early identification of insider activity. Three key predictions that the present study aims to test were that, during interview, insiders will use a significantly greater percentage of the following words compared to non-insiders: first-person singular pronouns (H1); words relating to cognitive processes (H2); and words relating to negative affect (H3). A final prediction concerns temporal changes in language use with respect to the Game Part (Part 1, Part 2, Part 3, Part 4), whereby language differences between insiders and non-insiders will become significantly more pronounced when interviewees are talking about later Game Parts relative to earlier Game Parts, reflecting the fact that insiders are likely to have become increasingly immersed in their insider activities from Game Part 2 onwards (H4).

## Method

### Design

A 2 (Interviewee Type: insider, non-insider) x 4 (Game Part: Part 1, Part 2, Part 3, Part 4) mixed design was employed. The dependent variables were three language categories: the pronoun ‘I’; negative emotion words; and words relating to cognitive processes.

### Data and procedure

The data for the study took the form of verbatim transcripts of 54 audio-visual recordings of tactical information-gathering interviews, collected as part of the Confidential Operations Simulation (see Dando et al., 2024, and Taylor et al., 2013). Dyadic interviews were conducted individually by one of four interviewers in a room in a university building. Interviews took place after the participants had completed all four parts of the simulation.

In brief, the simulation involved individuals working in teams to solve crimes. Part 1 of the game was a familiarisation phase that allowed participants to acquaint themselves with the gameplay and develop working relationships with one another. In Part 2 of the game, nine individuals (one person from each team) were approached at random and incentivised to act as insiders throughout the remaining game parts. They were covertly asked to complete tasks and provide the information to a provocateur for an additional £20 monetary incentive. As the insiders progressed through the game periods, the investigative tasks increased in complexity. In Part 2, insiders were asked to obtain and provide information that related to a person under investigation (information that was task-relevant to them and their team). In Part 3, insiders were asked to provide information from a database that their team had legitimate access to, but to which they had no direct access (information that was task-relevant to the team, but not to them). In Part 4, insiders were tasked to retrieve information from another team’s database to which the insider did not have legitimate access.

After completing Part 4 of the simulation, all participants were informed that there had been a security breach and that they would each be questioned, in turn, about their gaming behaviour during an interview. Prior to the interview, insiders were incentivised to deceive the interviewer by hiding their malicious activity and were given time to formulate a deceptive account. All interviews took place within two hours of each simulation game finishing.

### Interview protocol

Irrespective of condition (insider; non-insider), all tactical interviews were similarly structured and comprised the same four phases, in the same order (see Table 1). In Phase 1, participants were given the four ground rules. In Phase 2, participants were asked a series of set questions all centred on gaming behaviours designed to capture each participant’s version of their gaming ‘truth’. Leveraging an account of participants’ gaming behaviours early in the interview process allowed interviewers to use this account to accept or refute responses as the interview progressed and to note any inconsistencies across phases. In Phase 3, interviewees were asked four information-gathering questions. In Phase 4, interviewees were invited to ask questions and were offered the opportunity to alter anything they had previously said. At this point, the interview was drawn to a close (see Dando et al., 2018; Dando & Bull, 2011; Sandham et al., 2017). Although the protocol comprised four phases, interviewers moved through the phases in a seamless manner.

**Table 1.** Interview protocol.

Phase 1
Introduction to the ground rules:
Provide as much detail as possible. Tell me absolutely everything. Say if you cannot remember. I only want you to tell me what you actually remember. Do not guess. Tell me if you do not understand.
Phase 2
Deployment of a set of 20 standard gaming-behaviour questions:
1 Tell, Explain, or Describe (TED). 8 probing who, what, why, when, how questions (5 WHT questions). 11 closed questions (requiring a yes/no type response), designed to commit each interviewee to a version of their gameplay ‘truth’. Questions were centred on: Team membership and team remit. Individual role. Individual tasks. Database access (legitimate, attempted, successful, and illegitimate). Type of information held on legitimately accessed database. Communication behaviours (verbal, behavioural, electronic synchronous and asynchronous, and hardcopy).
Phase 3
Deployment of four information-gathering game recap questions using a TED invitation – one for each game:
Please explain to me what happened in Round 1, again. Please explain to me what happened in Round 2, again. Please explain to me what happened in Round 3, again. Please explain to me what happened in Round 4, again.
In Phase 3, the tactical approach was initiated whereby the interviewer probed the interviewee’s account of each round, in turn. Here the interviewer either accepted or refuted the interviewee’s answers, before moving on to the next question and without revealing to the interviewee any information that might be available to the interviewer until after the interviewee had answered the question. Where appropriate, the interviewer also questioned apparently erroneous responses. This tactical technique was possible because the interviewer could access the gameplay information via a tablet, where each player’s ‘footprint’ was presented in the form of a movement and behaviour timeline. The timeline data included the available game data for each player, which could then be used to accept or refute responses to each of the closed, probing or TED questions posed in Phase 1
Throughout Phase 2, clarification was sought from interviewees only where information was provided that was unclear, inconsistent, or contradicted previous responses. The interviewer was fully open with respect to referring to and/or indicating the availability of information on the tablet.
Phase 4
Offer the interviewee the opportunity to add to or alter anything that they had previously said.
Close the interview.

Throughout, the interviewer was able to access an individual player’s game activity (i.e. movements, interactions, and database access) via hand-held tablets as an aid to generate probing questions and signal inconsistencies between interviewee responses and automated game activity. Insider interviews ranged in length from 10.22 min to 52.00 min (*M* = 22.81 min, *SD* = 12.81) and the non-insider interviews ranged from 9.14 min to 38.31 min (*M* = 23.21 min, *SD* = 7.60).

### Interviewers

Four interviewers took part in the research (3 male and 1 female) aged between 42 and 56 years. Interviewers were serving or ex-police investigators with a minimum of 10 years’ experience of conducting information-gathering investigative interviews in the UK. Although interview protocols used for this research were investigative interviewing best-practice compliant, all interviewers underwent bespoke training over a two-day period, designed for this research by the fourth author, adopting a collaborative pedagogical approach, comprising: (i) a two-hour long classroom-based introduction to the interview protocol behaviours; and (ii) a two-hour long practice session that included three practice interviews, which were digitally recorded to allow for feedback and evaluation. Once the interviewers had attended the classroom training sessions (Training Day 1) and completed the practice interviews to a required level of competency (Training Day 2), they were able to commence research interviews. Importantly, interviewers were naïve as to whether interviewees were insiders or non-insiders.

### Adherence to the interview protocol

A random selection of 12 interviews (three conducted by each interviewer) were coded for interviewer adherence to the interview protocol (see Dando et al., 2024; Nahouli et al., 2023). Each interview was coded by two independent coders blind to the aims and hypotheses of the research. Coders scored each interview for presence of: (i) the four phases of the interview protocol; (ii) the ground rules from Phase 1; (iii) the 20 gaming-behaviour questions from Phase 2; and (iv) the four information-gathering questions from Phase 3. Each behaviour was coded as absent (scored 1), partially present (scored 2), and fully present (scored 3).

Prior to coding, coders participated in a training session held by the fourth author during which the interview protocols and the coding system were explained. Coders then practiced coding and discussed any disagreements/misunderstandings with the trainer to reach a consensus using a series of exemplar training interviews. Two-way mixed effects Intraclass Correlation Coefficient (ICC) analysis testing for absolute agreement between coders indicated very good inter-rater reliability for all interviewer behaviours: (i) the four phases, ICC = 1.000 (95% CI: 1.00; 1.00); (ii) the ground rules, ICC = 0.920 (95% CI: −0.449; 0.362); (iii) the 20 gaming-behaviour questions, ICC = 0.944 (95% CI: 0.889; 0.972); and (iv) the information-gathering questions, ICC = 1.000 (95% CI: 1.00; 1.00). Table 2 displays the mean adherence scores with respect to the interview protocol for each interviewer. Mean scores for each behaviour as a function of interviewer revealed a very high level of adherence to the interview protocol, with no significant differences across interviewers for each behaviour, all *F*s < 1.099, all *p*s > .879.

**Table 2.** Mean adherence scores (and standard deviations) with respect to the interview protocol for each interviewer.

	Mean (SD)			
	Four Phases	Ground Rules	Gaming-Behaviour Questions	Information-Gathering Questions
Interviewer 1	3.00 (0.00)	3.00 (0.00)	3.00 (0.00)	2.33 (0.58)
Interviewer 2	3.00 (0.00)	2.68 (0.58)	3.00 (0.00)	3.00 (0.00)
Interviewer 3	3.00 (0.00)	3.00 (0.00)	3.00 (0.00)	3.00 (0.00)
Interviewer 4	3.00 (0.00)	2.33 (0.58)	3.00 (0.00)	3.00 (0.00)

### Data transcription

Each of the interviews was transcribed verbatim using an online transcription service: Otter (https://otter.ai/welcome). Once transcribed, transcripts were checked against the original audio-visual clips for consistency and correctness. Each transcript followed a script-like format, organised by speaker (i.e. ‘interviewer’ and ‘interviewee’ followed by their speech). The time at which each speaker vocalised during the interview was indicated in minutes and seconds next to their label; for example, ‘Interviewer (09 min and 33 sec)’. Each transcription was subsequently split into four documents, categorised by each individual Game Part (Parts 1 to 4).

### Linguistic Inquiry and Word Count (LIWC)

Consistent with previous research (Richardson et al., 2014; Richardson & Nash, 2022; Tausczik & Pennebaker, 2010; Taylor et al., 2013), individual language use was examined by means of the computer software program LIWC (Pennebaker et al., 2007). LIWC analyses a text file on a word-by-word basis to calculate the total percentage of words that match various linguistic categories. The three categories of interest in this study were: the pronoun ‘I’, negative emotion words, and words relating to cognitive processes (Taylor et al., 2013). The 54 interview transcripts were split by Game Part (Part 1, Part 2, Part 3, Part 4), which resulted in 216 transcripts being produced (54 transcripts for each Game Part). Next, these 216 transcripts were split by speaker, namely interviewer and interviewee, to produce a total of 432 transcripts (216 interviewee transcripts and 216 interviewer transcripts). All 216 interviewee transcripts were then submitted to LIWC and analysed by Game Part. The resulting LIWC scores for each of the three categories present in Part 1 were compiled. This process was repeated for Parts 2, 3, and 4 and subsequently analysed in SPSS.

### Transparency and openness

We describe our sampling plan, all data exclusions (if any), all manipulations, and all measures in the study, and we adhered to the *Journal of Applied Psychology* methodological checklist. All data are available at [https://osf.io/jkbpm/?view_only=fc263915fb1c420db35bd8330b6006ce]. Data were analysed using SPSS Statistics, version 29.0. This study’s design and its analysis were not preregistered.

## Results and discussion

Descriptive statistics are presented in Table 3, which shows the mean percentage of occurrence of the pronoun ‘I’, negative emotion words, and words relating to cognitive processing across Interviewee Type (insider, non-insider) and Game Part (Part 1, Part 2, Part 3, Part 4).

**Table 3.** Data summary (mean percentages and standard deviations) for the effect of Interviewee Type (insider vs non-insider) and Game Part (Part 1, Part 2, Part 3, Part 4) on the relative frequency of language use.

Language Category	Interviewee Type	Game Part				
		Part 1	Part 2	Part 3	Part 4	Total
The Pronoun ‘I’	Insiders	10.27 (3.12)	4.60 (2.62)	8.10 (3.87)	13.56 (5.08)	9.13 (0.53)
	Non-insiders	5.17 (2.46)	5.72 (2.62)	5.32 (2.56)	4.91 (2.08)	5.28 (0.24)
	Total	6.05 (3.21)	5.53 (2.63)	5.80 (2.98)	6.41 (4.30)	
Negative Emotion Words	Insiders	0.37 (0.38)	0.35 (0.39)	0.68 (0.54)	1.68 (2.23)	0.77 (0.11)
	Non-insiders	0.43 (0.39)	0.40 (0.51)	0.31 (0.41)	0.61 (0.66)	0.44 (0.05)
	Total	0.42 (0.39)	0.39 (0.49)	0.37 (0.45)	0.79 (1.14)	
Words Relating to Cognitive Processing	Insiders	12.10 (3.18)	19.49 (6.92)	19.89 (5.58)	19.95 (3.29)	15.21 (0.76)
	Non-insiders	13.21 (2.77)	14.38 (3.79)	14.36 (3.72)	14.85 (3.44)	14.26 (0.35)
	Total	13.02 (2.85)	15.27 (4.81)	15.31 (4.56)	15.73 (3.90)	

### First-person singular pronouns

A 2 × 4 mixed design ANOVA, with Interviewee Type (insider vs non-insider) as the between-participants factor and Game Part (Part 1, Part 2, Part 3, Part 4) as the within-participants factor, was conducted to test H1, that insiders will use a significantly greater percentage of first-person singular pronouns when compared to non-insiders, and H4, that the language differences that are associated with insiders compared to non-insiders will become significantly more pronounced over time.

**Figure 1.**
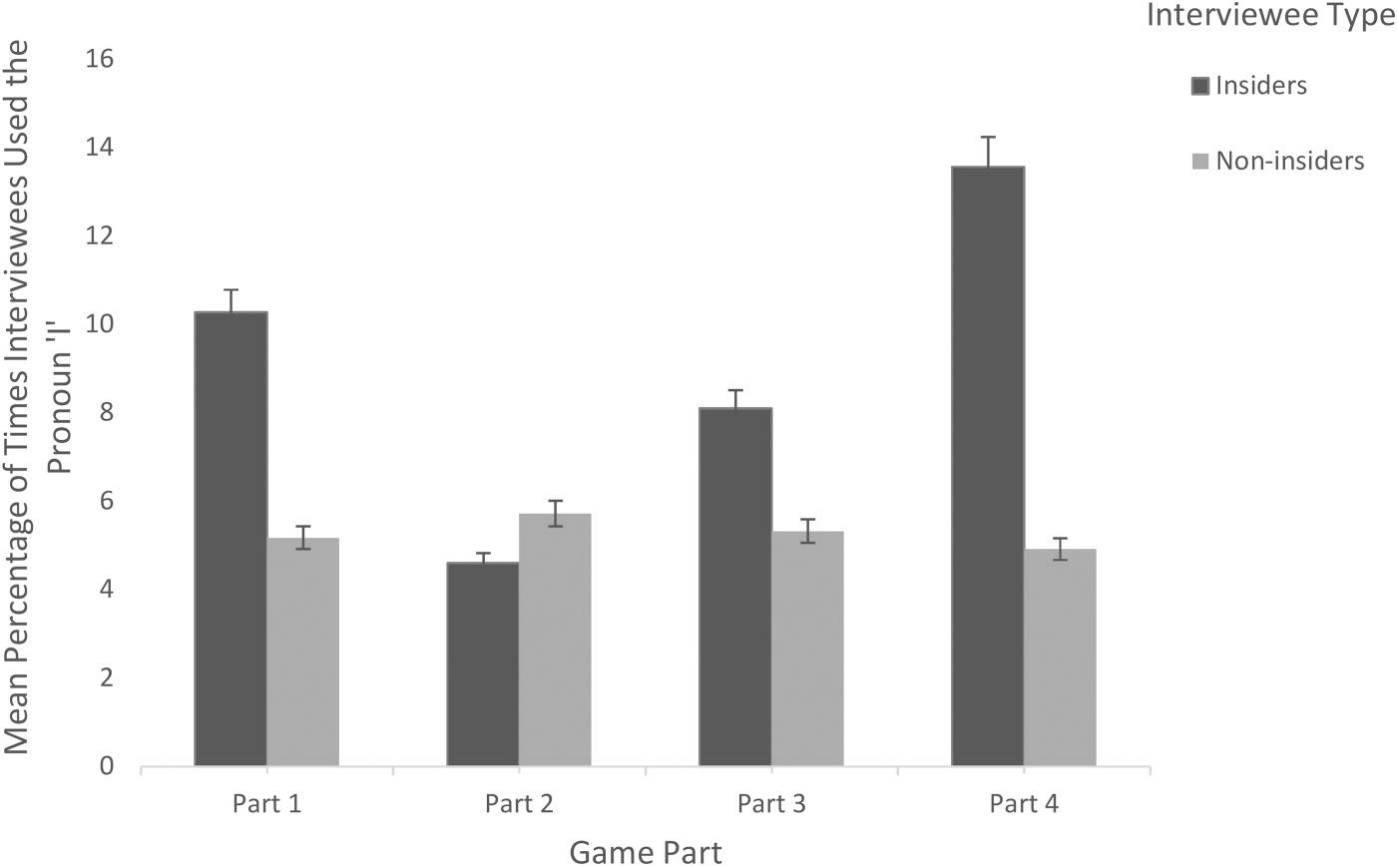
The mean percentage of times insiders and non-insiders used the pronoun ‘I’ when referring to each Game Part (the error bars represent 95% confidence intervals).

In relation to the use of first-person singular pronouns, there was a significant main effect of Interviewee Type on the percentage of times that ‘I’ was used by interviewees. Insiders (*M =* 9.13, *SD* = 0.53) used ‘I’ more frequently than non-insiders (*M =* 5.28, SD = 0.24), *F*(1, 50) = 43.36, *p* < .001, η_p_^2^ = 0.46, supporting H1. The main effect of Game Part was also significant; when participants were describing Part 4 (*M =* 6.41, *SD* = 4.30) they used the word ‘I’ more often than they did when describing Part 1 (*M =* 6.05, *SD* = 3.21), Part 2 (*M =* 5.53, SD = 2.63), and Part 3 (*M =* 5.80, SD = 2.98), *F*(3, 150) = 13.82, *p* < .001, η_p_^2^ = 0.21. A significant interaction also emerged between Interviewee Type and Game Part, *F*(3, 150) = 19.75, *p* < .001, η_p_^2^ = 0.28, with the data (see Figure 1) giving some indication that changes in the use of ‘I’ between insiders and non-insiders became more pronounced as insiders described Game Parts during which they had become increasingly immersed in insider activity, in line with H4.

To unpack this interaction, we first undertook simple main effects analyses of the data split by Interviewee Type (i.e. insiders vs non-insiders). For the insiders’ use of the pronoun ‘I’, the simple main effect of Game Part was statistically significant, *F*(3, 24) = 9.69, *p* < .001, η_p_^2^ = 0.55, whereas for non-insiders’ use of the pronoun ‘I’, the simple main effect of Game Part was not reliable, *F*(3, 126) = 0.97, *p* = .408, η_p_^2^ = 0.02. To understand the simple main effect of Game Part on insiders’ use of the word ‘I’, we conducted six paired-samples t-tests with a Bonferroni correction (corrected alpha = .008). Results revealed a significant difference between Part 1 and Part 2, *t*(8) = 3.59, *p* = .007, with insiders who were talking about Part 1 (*M =* 10.27, *SD =* 3.12) using ‘I’ pronouns more than when they were describing Part 2 (*M =* 4.60, *SD =* 2.62). A significant difference was also found between Part 2 and Part 4, *t*(8) = −4.97, *p* < .001, with insiders who were talking about Part 2 (*M =* 4.60, *SD =* 2.62) using ‘I’ less than when talking about Part 4 (*M =* 13.56, *SD =* 5.08). No significant difference was found for data relating to Part 1 versus Part 3, *t*(8) = 1.48, *p* = .178, Part 1 versus Part 4, *t*(8) = −1.45, *p* = .185, Part 2 versus Part 3, *t*(8) = −3.05, *p* = .016, or Part 3 versus Part 4, *t*(8) = −3.05, *p* = .016. These analyses provide some support for H4, inasmuch as there was evidence for insiders’ use of the pronoun ‘I’ being significantly heightened when they were referring to Game Part 4 (i.e. the point in the game when they had been immersed in complex insider activity), relative to when they were referring to Game Part 2 (i.e. when they had first engaged in low-level insider activity).

To unpack fully the interaction between Interviewee Type and Game Part, four independent samples t-tests with a Bonferroni correction (corrected alpha = .0125) were also conducted to compare the use of the word ‘I’ by insiders and non-insiders when they were referring to each Game Part. There was a significant difference in the number of times the word ‘I’ was used by interviewees when referring to Part 1 of the game, *t*(50) = −5.40, *p* < .001, with insiders (*M =* 10.27, *SD =* 3.12) using the word ‘I’ more than non-insiders (*M =* 5.17, *SD =* 2.46). There was also a significant difference between insiders and non-insiders when referring to Part 3, *t*(50) = −2.70, *p* = .005, with insiders (*M =* 8.10, *SD =* 3.87) using the word ‘I’ more than non-insiders (*M =* 5.32, *SD =* 2.56). A further significant difference was revealed for interviewees referring to Part 4, *t*(50) = −8.46, *p* < .001, with insiders (*M =* 13.56, *SD =* 5.08) using the word ‘I’ more than non-insiders (*M =* 4.91, *SD =* 2.08). There was no significant difference in the use of the word ‘I’ between insiders and non-insiders when referring to Part 2 of the game, *t*(50) = 1.17, *p* = .124. These results provide further support for H1.

### Negative emotion words

A 2 × 4 mixed design ANOVA, with Interviewee Type (insider vs non-insider) as the between-participants factor and Game Part (Part 1, Part 2, Part 3, Part 4) as the within-participants factor, was conducted to test H2, that insiders will use a significantly greater percentage of negative emotion words than non-insiders, and H4, that the language differences that are associated with insiders compared to non-insiders will become significantly more pronounced when they are referencing later versus earlier Game Parts. This analysis revealed a significant main effect of Interviewee Type on the number of negative emotion words used by interviewees, with insiders (*M =* 0.77, *SD* = 0.11) using negative emotion words more than non-insiders (*M =* 0.44, *SD* = 0.05), *F*(1, 50) = 7.18, *p* = .010, η_p_^2^ = 0.13, supporting H2. The main effect of Game Part was also significant; participants who were referring to Part 4 (*M =* 0.79, *SD* = 1.14) used words that related to negative emotions more frequently than when they were referring to Part 1 (*M =* 0.42, *SD* = 0.39), Part 2 (*M =* 0.39, *SD* = 0.49), and Part 3 (*M =* 0.37, *SD* = 0.45), *F*(3, 150) = 9.14, *p* < .001, η_p_^2^ = 0.16.

**Figure 2.**
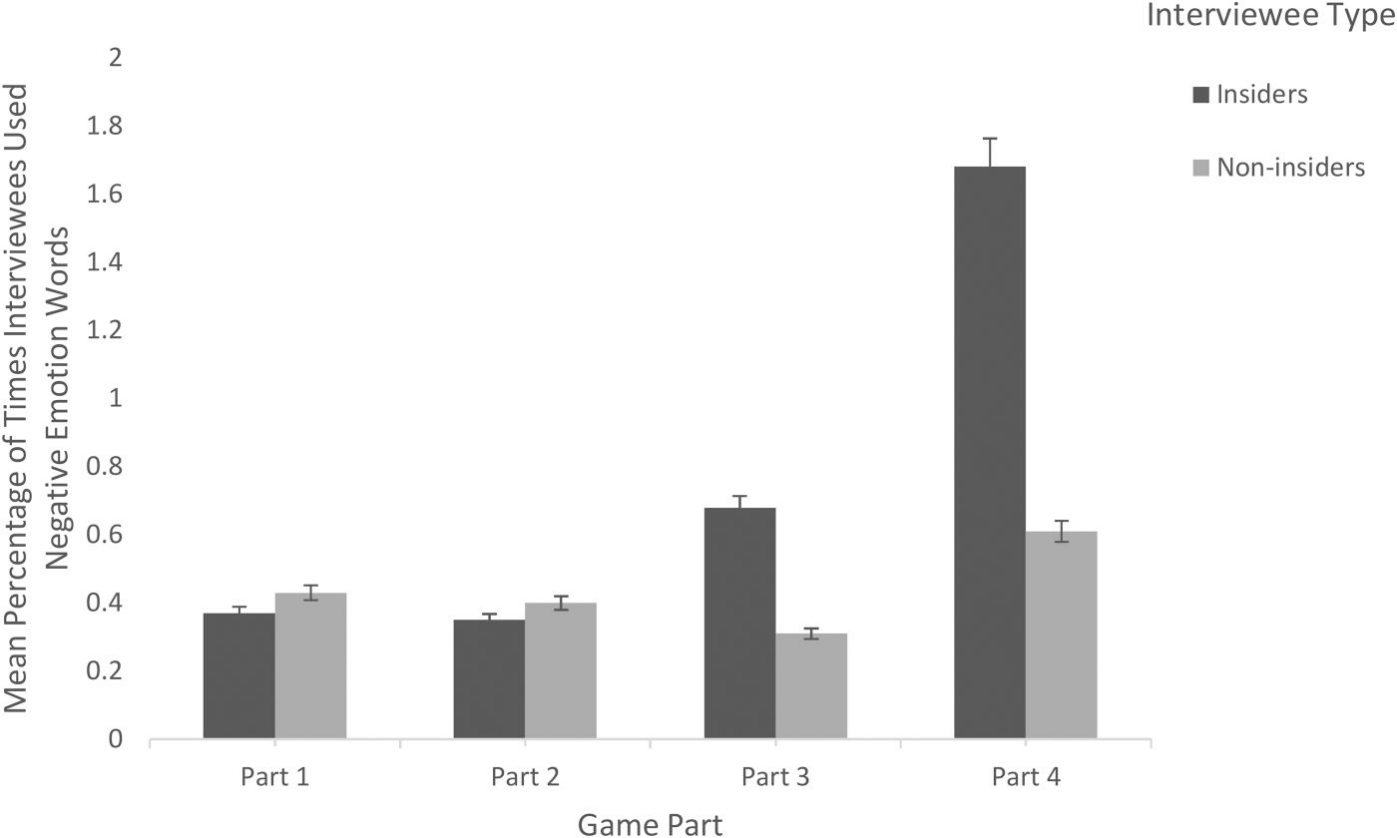
The mean percentage of times insiders and non-insiders used words relating to negative emotions when referring to each Game Part (the error bars represent 95% confidence intervals).

A significant interaction also emerged between Interviewee Type and Game Part, *F*(3, 150) = 4.98, *p* = .011, η_p_^2^ = 0.09, with the pattern of descriptive data again supporting H4 (see Figure 2). Two repeated-measures ANOVAs were conducted to investigate the interaction between Interviewee Type and Game Part, with data split by Interviewee Type. For insiders, there was no significant simple main effect of Game Part for the relative frequency of use of negative emotion words, *F*(3, 24) = 2.59, *p* = .141, η_p_^2^ = 0.24. Likewise, the simple main effect for non-insiders was not significant, *F*(3, 126) = 2.64, *p* = .063, η_p_^2^ = 0.06. To explore further the basis of the significant interaction, we conducted independent samples t-tests with a Bonferroni correction (corrected alpha = .0125). There was no significant difference in the number of times negative emotion words were used by interviewees when referring to Part 1, *t*(50) = 0.47, *p* = 0.321, or Part 2 of the game, *t*(50) = 0.27, *p* = .393. However, when referring to Parts 3 and 4, insiders used significantly more negative emotion words than non-insiders, thereby supporting H4: Part 3, *t*(50) = −2.34, *p* = .012; Part 4, *t*(50) = −2.73, *p* = .004.

### Words relating to cognitive processes

To test H3, that insiders will use a significantly greater percentage of words relating to cognitive processes than non-insiders, as well as H4, a 2 × 4 mixed design ANOVA was conducted, with Interviewee Type (insider vs non-insider) as the between-participants factor and Game Part (Part 1, Part 2, Part 3, Part 4) as the within-participants factor. This revealed no significant main effect of Interviewee Type on the number of times words related to cognitive processing were used by interviewees, *F*(1, 50) = 1.28, *p* = .263, η_p_^2^ = 0.03. However, the main effect of Game Part was significant, *F*(3, 150) = 13.21, *p* < .001, η_p_^2^ = 0.21.

**Figure 3.**
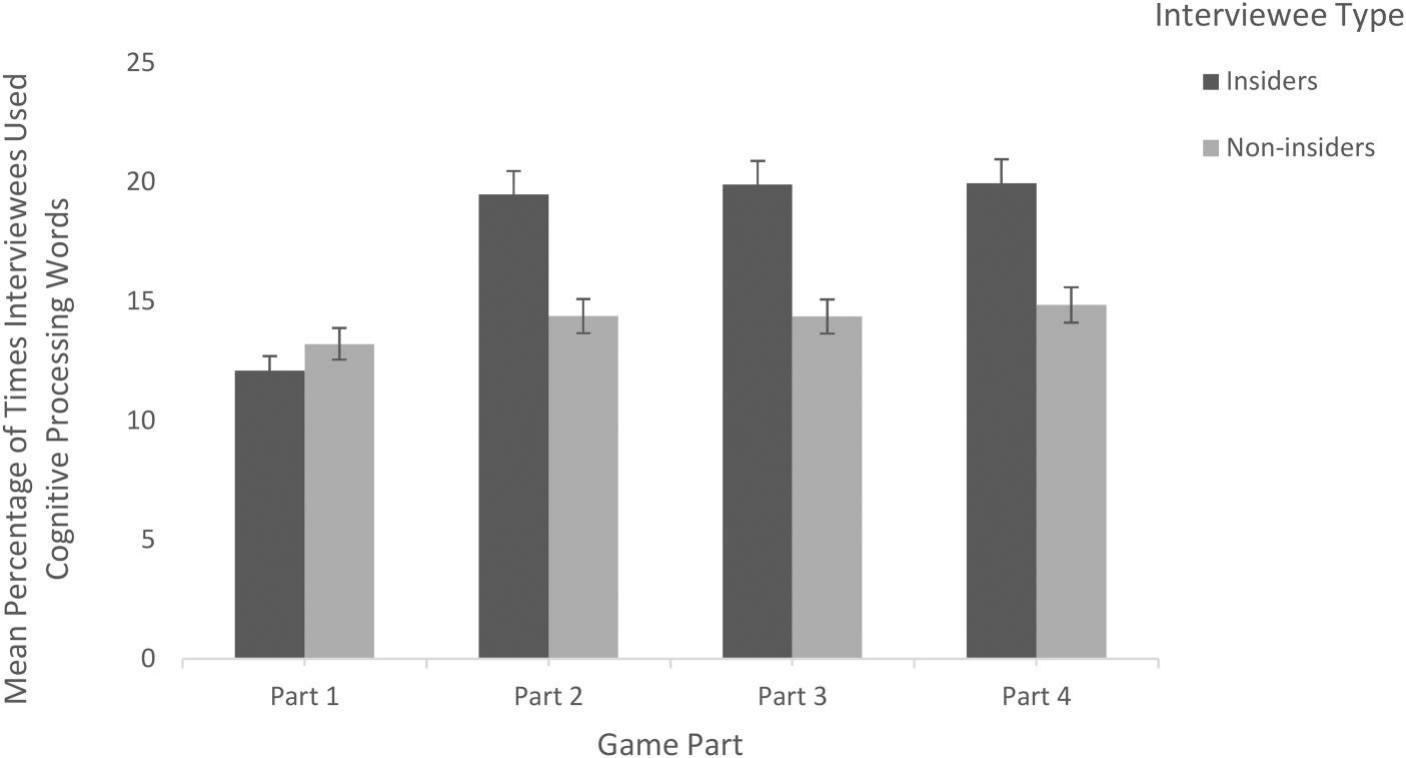
The mean percentage of times insiders and non-insiders used words relating to cognitive processing when referring to each Game Part (the error bars represent 95% confidence intervals).

A significant interaction also emerged between Interviewee Type and Game Part, *F*(3, 150) = 6.91, *p* < .001, η_p_^2^ = 0.12. The pattern of data (Figure 3) provides some support for H4, in that the use of words referring to cognitive processing was heightened for insiders relative to non-insiders from Part 2 of the game onward, that is, from the first point at which insiders were tasked with engaging in insider activity. Two repeated-measures ANOVAs were conducted to investigate the interaction between Interviewee Type and Game Part, with data split by Interviewee Type. For insiders, there was a significant simple main effect of Game Part on the relative frequency with which cognitive processing words were used, *F*(3, 24) = 8.20, *p* = .004, η_p_^2^ = 0.51. For non-insiders, the simple main effect of Game Part was not significant, *F*(3, 126) = 2.40, *p* = .071, η_p_^2^ = 0.05.

To further understand the simple main effect of Game Part on insiders’ use of words relating to cognitive processing, we conducted six paired-samples t-tests with a Bonferroni correction (corrected alpha = .008). Results revealed a significant difference in the relative frequency with which cognitive processing words were used by insiders when referring to Part 1 versus Part 2 of the game, *t*(8) = −4.75, *p* < .001, with insiders who were talking about Part 1 (*M =* 12.10, *SD* = 3.18) using words relating to cognitive processing less than when they were talking about Part 2 (*M* = 19.49, *SD* = 6.92). Similarly, significant differences were also found between Part 1 and Part 3 (*M =* 19.89, *SD* = 5.58), *t*(8) = −4.33, *p* = .002, and between Part 1 and Part 4 (*M =* 19.95, *SD =* 3.29), *t*(8) = −6.56, *p* < .001. However, paired-samples t-tests revealed no significant difference between Part 2 and Part 3*, t*(8) = −0.21, *p* = .841, between Part 2 and Part 4, *t*(8) = −0.18, *p* = .861, or between Part 3 and Part 4, *t*(8) = −0.03, *p* = .981.

To unpack fully the interaction between Interviewee Type and Game Part, four independent samples t-tests with a Bonferroni correction (corrected alpha = .0125) were conducted to compare the use of words relating to cognitive processing by insiders and non-insiders when referring to each Game Part. There was no significant difference in the relative frequency with which cognitive processing words were used by insiders and non-insiders when talking about Part 1 of the game, *t*(50) = 1.07, *p* = .146. However, significant differences between insiders and non-insiders were observed in relation to Part 2, *t*(50) = −3.14, *p* = .001, Part 3, *t*(50) = −3.71, *p* < .001, and Part 4, *t*(50) = −4.07, *p* < .001, with insiders (Part 2: *M =* 19.49, *SD =* 6.92; Part 3: *M =* 19.89, *SD =* 5.58; Part 4: *M =* 19.95, *SD =* 3.29) using words relating to cognitive processing more than non-insiders (Part 2: *M =* 14.38, *SD =* 3.79; Part 3: *M =* 14.36, *SD =* 3.72; Part 4: *M =* 14.85, *SD =* 3.44).

### Insider Language Index

The findings that these three language categories (pronoun use, negative affect, and cognitive processing) can differentiate insiders from their non-insider counterparts, suggest that language analysis could offer a useful means of detecting insider activity. To explore this idea, we created a composite measure to detect insider threat through language change, in the form of an ILI for each participant for each Game Part. We did this by first calculating a standardised *Z*-score for each participant’s language measure in each category. Next, these scores were aggregated into a composite ILI for each participant at each Game Part. Higher ILI scores are interpretable as reflecting a greater overall use of ‘insider language’ by an interviewee when describing their activity. As a composite language measure, we anticipated that the ILI would have the potential to distinguish insiders from non-insiders with a high degree of reliability.

To test the capacity of the ILI to identify insiders relative to non-insiders, we conducted a 2 × 4 mixed design ANOVA with ILI scores as the dependent measure, Interviewee Type (insider, non-insider) as the between-participants factor and Game Part (Part 1, Part 2, Part 3, Part 4) as the within-participants factor. This analysis revealed a significant main effect of Interviewee Type on ILI scores, *F*(1, 50) = 23.14, *p* < .001, η_p_^2^ = 0.32, with insiders (*M* = 1.82) demonstrating higher ILI scores than non-insiders (*M* = −0.38). The main effect of Game Part was also significant, *F*(3, 150) = 4.00, *p* = .009, η_p_^2^ = 0.07. Additionally, a significant interaction was observed between Interviewee Type and Game Part, *F*(3, 150) = 9.35, *p* < .001, η_p_^2^ = 0.16.

**Figure 4.**
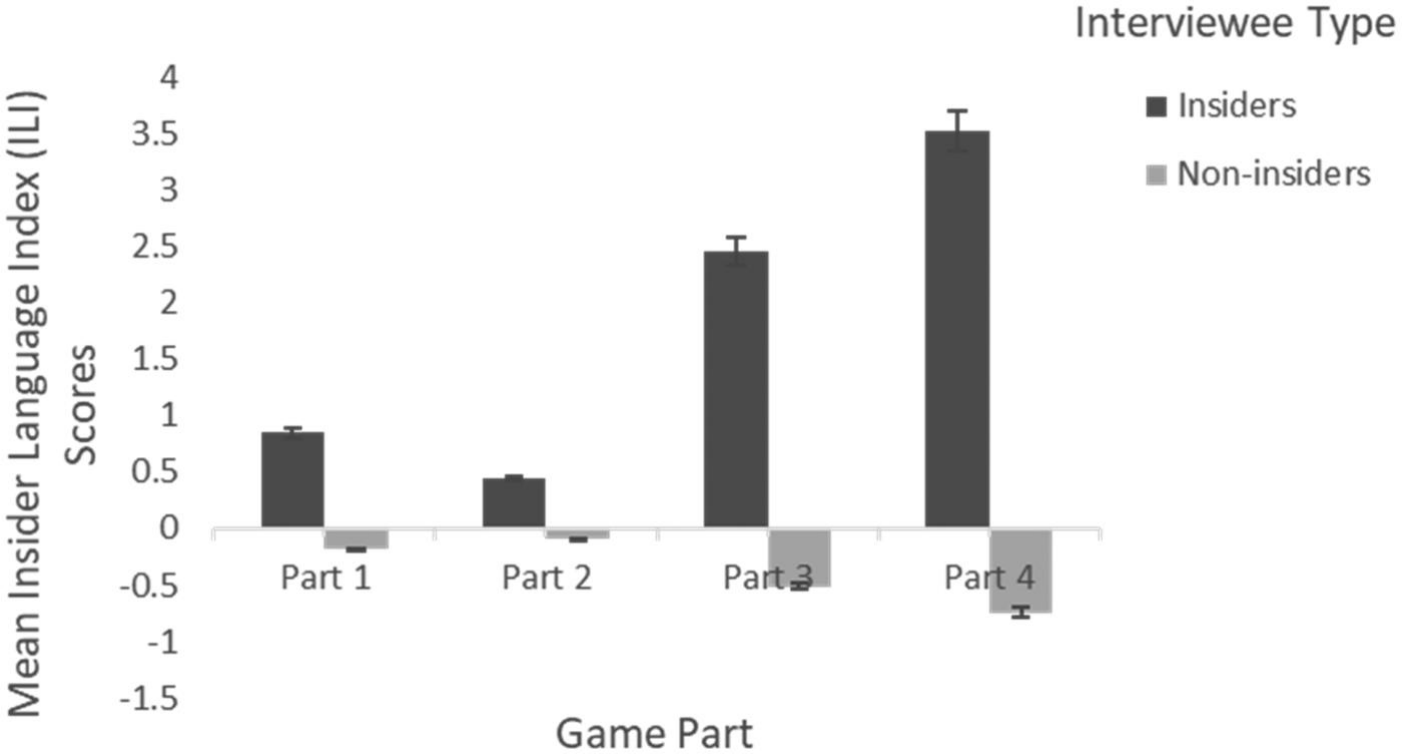
The mean Insider Language Index (ILI) scores for insiders and non-insiders in relation to each Game Part (the error bars represent 95% confidence intervals).

Two repeated-measures ANOVAs were conducted to investigate the interaction between Game Part and interviewee type when measuring ILI scores, with data split by Interviewee Type (Figure 4). These analyses revealed a significant simple main effect of Game Part on ILI scores for insiders, *F*(3, 24) = 7.55, *p* < .001, η_p_^2^ = 0.49, but no significant simple main effect for non-insiders, *F*(3, 126) = 1.63, *p* = .187, η_p_^2^ = 0.04. Six paired-samples t-tests with a Bonferroni correction (corrected alpha = .008) were conducted to compare insiders’ ILI scores across different Game Parts. There was no significant difference in insiders’ ILI scores for Part 1 versus Part 2, *t*(8) = 0.92, *p* = .387, or for Part 1 versus Part 3, *t*(8) = −42.52, *p* = .036. There was, however, a significant difference in insiders’ ILI scores for Part 1 versus Part 4, *t*(8) = −3.47, *p* = .008, with insiders who were describing Part 1 of the game demonstrating lower ILI scores (*M =* 0.85, *SD =* 1.74) than when they were describing Part 4 (*M =* 3.52, *SD =* 2.50). A significant difference in insiders’ ILI scores was also found between Part 2 and Part 3, *t*(8) = −4.39, *p* = .002, with insiders who were describing Part 2 of the game (*M* = 0.44, *SD* = 2.27) demonstrating lower ILI scores than when they were describing Part 3 (*M =* 2.46, *SD* = 3.04). A further paired-samples t-test also revealed a significant difference between insiders’ ILI scores for Part 2 versus Part 4 of the game, *t*(8) = −3.67, *p* =.006, with insiders demonstrating lower ILI scores when describing Part 2 of the game (*M* = 0.44, *SD* = 2.27) than when describing Part 4 (*M =* 3.52, *SD =* 2.50). A final paired-samples t-test revealed no significant difference between insiders ILI scores for Part 3 versus Part 4, *t*(8) = −1.00, *p* = .349.

To explore fully the interaction between Interviewee Type and Game Part, four independent samples t-tests with a Bonferroni correction (corrected alpha = .0125) were conducted to compare the ILI scores of insiders and non-insiders in relation to each Game Part. There were no significant differences between the ILI scores of insiders and non-insiders in relation to Part 1 of the game, *t*(50) = −1.64, *p* = .107, or Part 2, *t*(50) = −0.77, *p* = .445. However, there was a significant difference between the ILI scores of insiders and non-insiders in relation to Part 3 of the game, *t*(50) = −2.82, *p* = .019, with insiders (*M =* 2.46, *SD =* 3.04) demonstrating higher ILI scores than non-insiders (*M =* −0.51, *SD =* 1.93). There was also a significant difference between the ILI scores of insiders and non-insiders in relation to Part 4 of the game, *t*(50) = −4.99, *p* < .001, again with insiders (*M =* 3.52, *SD =* 2.50) demonstrating higher ILI scores than non-insiders (*M =* −0.74, *SD =* 1.23).

## General discussion

To date, most of the research into insider threat detection has used technological approaches to reduce the direct impact (e.g. financial loss, compromised data, damage to critical infrastructure) that an organisation may encounter because of an attack (Fenstermacher et al., 2022). These techniques typically identify insider activity based on access to unauthorised system information, which can limit their applicability to other situations. The present research instead took a behaviour-centric approach to the identification of insiders, by focusing on the human at the heart of insider activity. In adopting this approach, the key aim was to determine the potential value of analysing language use as a means of supporting the early detection of insider activity as part of a defensive strategy against these threats. Specifically, we build on the proposal advanced by Taylor et al. (2013) that the language used by insiders and non-insiders is different, and that this difference can be helpful in the detection of insider attacks.

Taylor et al. (2013) investigated the language used by insiders and non-insiders during email communication when participants were engaged in a team-based workplace simulation. They observed that, over time, insiders tended to become more self-focused, distancing themselves from their co-workers. This was reflected in their greater use of the ‘I’ pronoun. Insiders also showed increased use of negative emotion words and words related to cognitive processing. As we noted earlier, however, one concern with this evidence arises from the narrow focus of this previous study on the email communication between team members, who may have demonstrated language differences between insiders and non-insiders that do not extend to other situations, such as face-to-face investigative questioning. This kind of tactical interviewing might arise in an organisational context when a suspected insider is being asked by a company manager or IT security officer about suspicious behaviours. To test whether these language patterns hold across different contexts, we analysed post-simulation interview data from Taylor et al. (2013) that has not previously been analysed.

Using LIWC, we analysed the language used by insiders and non-insiders across our three language categories of interest, first identified by Taylor et al. (2013), namely, personal pronouns, negative affect, and cognitive processing. Our findings offer empirical support for H1; insiders used the word ‘I’ significantly more often than their non-insider counterparts in the post-simulation investigative interviews. Similar findings have also been reported by Kacewicz et al. (2014), who showed that individuals who are self-focused tend to use a greater proportion of personal pronouns, as compared with individuals who are more collectively orientated and externally focused. The association between language use and a change in self-focus can be applied to the context of insider activity, where there is a shift from collaborative work to individual activity that is linked to the insider attack. The current findings indicate that even after an attack has occurred, the language of insiders remains more self-focused and less team-orientated than their co-workers in an interview context.

The findings of the current study also provide empirical support for H2; insiders used significantly more negative emotion words than their non-insider counterparts during the post-simulation interviews. Such findings are consistent with research on verbal cues to deceit, which has shown that deception is often associated with an increase in negative emotion words (Newman et al., 2003). Research suggests that this increase occurs because deceivers feel guilty (Vrjj, 2000). The idea that deceivers use more words related to negative emotion aligns with Taylor et al.’s (2013) findings that during an insider attack, insiders expressed more negative emotion than non-insiders. Again, the current study extends this research and indicates that such language differences relating to negative affect remain present even after an attack has occurred.

Additionally, the findings from the current study demonstrate support for H3; insiders used words related to cognitive processing significantly more often than their non-insider counterparts when being interviewed. Such findings are again consistent with the literature on deception detection, which has found that deceivers show increased signs of cognitive load compared to their honest counterparts (Blandón-Gitlin et al., 2014) and this can manifest as an increase in words related to cognitive processing. This research indicates that deceivers experience increased cognitive effort because they must simultaneously monitor the interviewer’s reaction to their deception to assess the extent to which they are believed (Vrij et al., 2008). Additionally, research suggests that when individuals engage in deception, they engage in impression management to avoid displaying cues to deceit, which requires extra cognitive effort (Vrij et al., 2008).

Finally, the present findings also support H4, whereby we find that the language differences that we have identified between insiders and non-insiders become significantly more pronounced when interviewees are questioned about the activities that they engaged in during later parts of the simulation (i.e. Game Parts 3 and 4) compared to the earlier parts. In Game Parts 3 and 4, insiders had been tasked to complete increasingly complex insider activities (e.g. to access another employee’s database for information). They received a further monetary incentive during these game parts for engaging in additional insider tasks. Our findings based on the analysis of the post-simulation interviews indicate that when insiders were questioned about their previous activities in each game part, they demonstrated a change in language use that mirrored that found in their email communication during the simulation itself. This finding presumably reflects the fact that when answering interview questions about their activities in Game Parts 3 and 4, insiders were likewise engaged in heightened levels of deception to conceal from the interviewer what their true intentions had been during these points in the simulation, where they were increasingly immersed in the attack.

Overall, our results suggest that insiders have a distinctive language pattern that differs from non-insiders and that knowledge of this might be useful in aiding the detection of insider threat. The unique language pattern shown in our results (i.e. increased use of personal pronouns and words relating to negative affect and cognitive processing), encouraged us to develop an ILI as a composite measure of language change for use in insider threat detection. As predicted, insiders had a higher ILI score than non-insiders, signifying the potential value of the ILI to distinguish between insiders and non-insiders with a high degree of reliability. The way that the ILI is calculated through the standardisation of language scores across three linguistic categories and the subsequent aggregation of these scores for each person, meant that it provided a unique index of the overall use of ‘insider language’ by an interviewee when describing their activity. The finding that even during a post-attack interview the ILI can differentiate between insiders and non-insiders suggests that this measure could be generalisable and valuable for assisting organisations in detecting insiders to prevent additional attacks from being conducted. This would be particularly helpful given that insiders often conduct multiple attacks prior to being detected (Pfleeger, 2008).

### Limitations and future directions

To understand more fully the validity of our ILI measure, we need to consider whether patterns of language use are transferable to real-world organisational contexts. Therefore, analysing findings from multiple insider threat studies, perhaps utilising a similar methodology across a greater number of contexts, would give a more generalisable picture of the language used by insiders and how this could be identified by employers early on to mitigate the financial loss and disruption that can be caused because of an insider attack. It is encouraging to note that the language differences that we have identified are found both during an attack (Taylor et al., 2013) and in post-attack interviews. ILI patterns in our research were also found to be present across different interviewers, suggesting that language changes are stable to the individual conducting the insider attack, rather than being a feature of the dynamic interaction between interviewer and interviewee (Richardson et al., 2019).

It is also important, however, to treat our ILI measure with caution in terms of its application within real-world organisations. The ILI should not be viewed as a diagnostic tool to identify insiders with certainty, but rather as an approach to alert managers or security staff to the possibility that an employee is showing a distinctive pattern of language use that could warrant further investigation. It may be the case, for example, that insiders show similar patterns of language use to disgruntled employees (e.g. ones who have failed to receive an expected promotion) but who have not engaged in malicious insider activity. The similarities, or differences, between the language of such disgruntled employees and insiders remain to be tested, as well as the nature of the language changes that arise if an increasingly disgruntled individual transitions over time to become an insider by engaging in malicious activities aimed at damaging a company.

Although our results suggest the potential importance of language use as a means to detect insider threat, there is much scope for further research to explore these effects to a greater extent. For example, it is not yet clear whether these language differences could help in the identification of insiders by human observers (e.g. by an interviewer) in the absence of a formal language analysis using LIWC. In addition, only the interview transcripts were analysed in the current study. These transcripts allowed us to test our hypotheses relating to language use, but we may have missed other important insider behaviours that are only identifiable through different types of interview recordings. For example, audio and visual formats may contribute to a focus on potentially significant non-verbal cues, such as tone of voice or fidgeting, which could be perceived by an observer to signal deception (Driskell, 2012) and, in turn may prove to be important when attempting to identify suspicious activity (Bond & DePaulo, 2006). Therefore, future research should aim to investigate insider threat detection further by employing multiple interview formats to explore how accurate individuals are at detecting insider activity across different media.

## Conclusion

This study provides further support for the notion that individual language use is a crucial factor in the detection of insider activity. This research significantly extends the previous literature by developing the first index that measures language features unique to insider activity. Theoretically, this advances our understanding of the extent to which language can tell us important things about individual cognition and motivation. Practically, it opens up the potential for the development of language-based models of malintent that can be used in early detection of insider threat. This early detection will help organisations prevent further losses.
